# Integrated analysis toolkit for dissecting whole‐genome‐wide features of cell‐free DNA

**DOI:** 10.1002/ctm2.1212

**Published:** 2023-02-28

**Authors:** Jie Li, Xin Sun, Heli Yang, Jiahui Chen, Zhaode Bu, Jiafu Ji, Xun Lan

**Affiliations:** ^1^ Department of Basic Medical Sciences School of Medicine, Tsinghua University Beijing China; ^2^ Key Laboratory of Carcinogenesis and Translational Research (Ministry of Education/Beijing) Center of Gastrointestinal Cancer, Peking University Cancer Hospital & Institute Beijing China; ^3^ Peking‐Tsinghua‐NIBS Joint Graduate Program Tsinghua University Beijing China; ^4^ Tsinghua‐Peking Joint Center for Life Sciences Tsinghua University Beijing China; ^5^ Center of Gastrointestinal Cancer Peking University Cancer Hospital & Institute, No. 52 Fucheng Road, Haidian District Beijing China; ^6^ MOE Key Laboratory of Bioinformatics Tsinghua University Beijing China

Dear Editor,

Patterns in whole‐genome‐wide features of cell‐free DNA (cfDNA) in human plasma provide a noninvasive diagnostic approach for cancer detection.^1^ However, an integrated analysis toolkit for whole‐genome‐wide features of cfDNA (INAC) is lacking. In this study, these cfDNA whole‐genome features, including cfDNA quality control, fragment size,^2^ fragment size ratio,^3^ copy number variation,^4^ transcription start site (TSS) relative coverage,^5^, ^6^ and promoter fragmentation entropy,^7^ were estimated by using the INAC toolkit to analyze a collected independent dataset (50 patients with gastric cancer and 50 healthy controls) with ∼10x sequence depth. Several functions are enabled in INAC to facilitate reproducible research (Table S1). The INAC was deposited in the Git hub: https://github.com/jacklee2thu/INAC.

Some accidents such as bumpy transport or poor storage conditions produced the hemolysis samples. Therefore, we simulate the hemolysis samples that are stored in EDTA tubes at room temperature for two hours and shake the tubes until observing red liquid induced by red blood cells ruptured. The hemolysis, healthy, and gastric cancer samples cfDNA counts of total reads, deduplicated reads, and human genome mapped reads were not significantly different after reads quality control (Figure S1A). However, most of the cfDNA reads from the hemolysis samples were longer than 150 bp compared to others (Figure 1A). More specifically, the 30–60 bp cfDNA read count from the hemolysis samples was greater than those from the other two types of samples, but the 60–80 bp cfDNA count was lower (Figure 1B). Starting from 150 bp, the hemolysis sample cfDNA count increased steeply (Figure 1C,D). In addition, the hemolysis samples had a lower cfDNA read fraction of 30 bp – 80 and 1000 bp—longer (Figure 1E). INAC would remind the users that the sample is at high risk of hemolysis when the cfDNA fragment size ratio is lower than 0.05. For the cfDNA quality control of individual samples, INAC indicated the read parameters in 24 chromosomes (Figure S1B–E).

**FIGURE 1:**
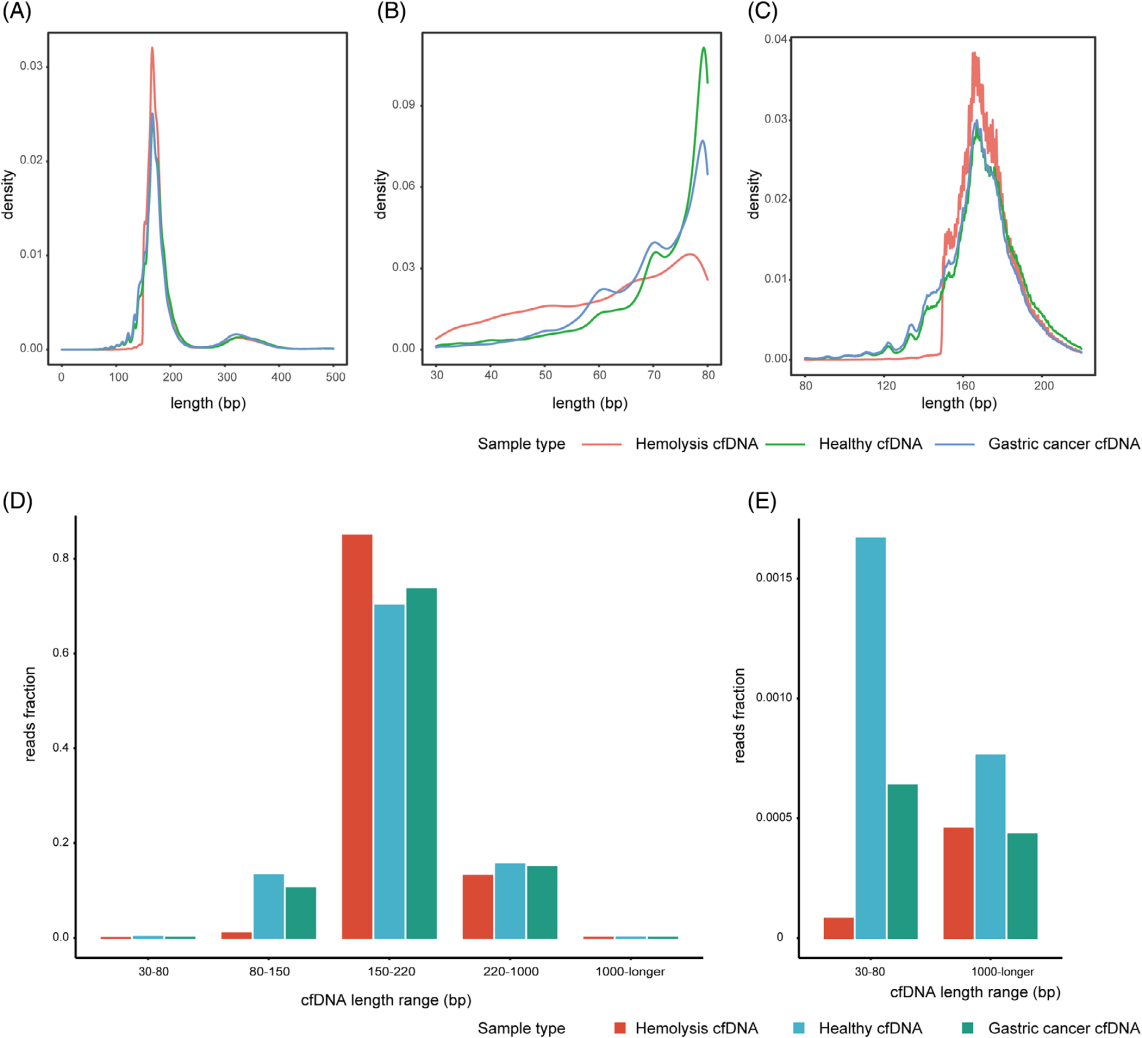
Integrated analysis toolkit for whole‐genome‐wide features of cell‐free DNA (INAC) quality control assessment of the hemolysis sample. (A–C) Density plot showing the cfDNA fragment sizes in the hemolysis, healthy and gastric cancer samples from 0 to 500 bp (A), 30–80 bp (B), and 80–220 bp (C). (D) The cfDNA read fraction was distinct in the 30–80 bp, 80–150 bp, 150–220 bp, 220–1000 bp, and 1000 bp—longer ranges in the hemolysis, healthy and gastric cancer samples. (E) More detailed cfDNA read fractions of 30–80 and 1000 bp—longer are displayed in the hemolysis, healthy and gastric cancer samples.

More variable and higher cfDNA fragment ratios in the blood of gastric cancer patients (0.07747539–0.63051678) were observed than in healthy individuals (0.05973131–0.26309905) (Figure 2A). After the fragment ratio was normalized, strong cfDNA normalized fragment ratio consistency appeared in healthy blood. However, it was heterogeneous in gastric cancer blood (Figure 2B,D,F). Another three cfDNA fragment size features, including the fraction of G and C‐corrected fragments, GC‐corrected short fragments (100‐150 bp), and GC‐corrected long fragments (150–220 bp), also showed similar performance (Figure 2C,E,G and Figure S2A–F).

**FIGURE 2:**
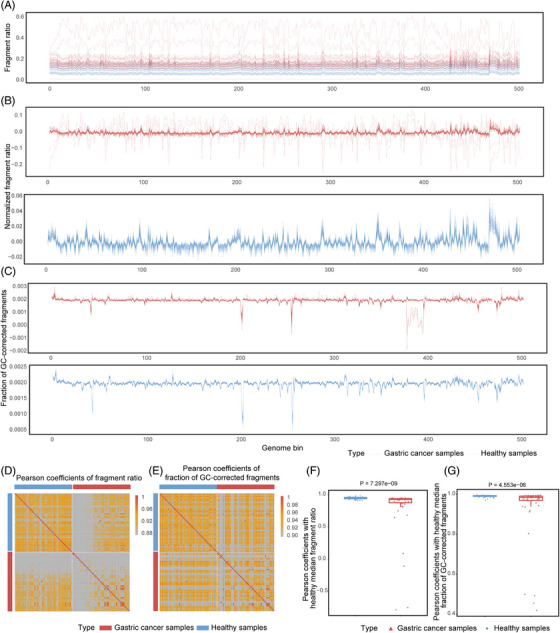
Integrated analysis toolkit for whole‐genome‐wide features of cell‐free DNA (INAC) fragment size ratio reveals the occurrence of cancer. (A) Original fragment ratios (defined as the ratio of short to long fragments in 5 Mb bins) are described for gastric cancer samples (*n* = 50) and healthy samples (n = 50). (B) Normalized fragment ratios (scale: mean fragment ratio = 0) are described for gastric cancer samples and healthy samples. (C) The fraction of GC‐corrected fragments (defined as the fraction of fragments in the 5 Mb bin to all fragments in all bins) is described for gastric cancer samples and healthy samples. (D‐E) The Pearson coefficients of the fragment ratio (D) and the fraction of GC‐corrected fragments (E) showing the similarity of individuals from gastric cancer samples and healthy samples. (F) Boxplot showing the fragment ratio Pearson coefficients between the healthy median fragment ratio and each other sample. (G) Boxplot showing the fraction of GC‐corrected fragment Pearson coefficients between the healthy median fraction of GC‐corrected fragments and each other sample. The two‐sided Wilcoxon test was used for the analysis.

In healthy individuals, the copy number variance (CNV) scores of most bins were concentrated around zero, whereas the variance in the CNV scores in patients with gastric cancer was larger (Figure S3A,B and Figure S4A, *p* = 0.023). Subsequently, significantly amplified CNV genes (n = 332) in gastric cancer patients were used to explore tumour‐derived programs (Figure S3C,D). Concretely, INAC identified the amplified CNV genes in tumour‐derived programs (Figure S4B).

To explore the relationship between promoter region cfDNA coverage and the corresponding chromatin state, cfDNA conventional relative coverage, cfDNA TSS NDR relative coverage and TSS 2K region relative coverage were used to estimate the promoter chromatin state (Figure 3A and Figure S5A). The genes with both TSS NDR relative coverage and TSS 2K region relative coverage less than 1 in more gastric cancer patients were considered to possess an open chromatin state (Figure 3B; red dots indicate permissive genes with an open TSS chromatin state). Whereas the genes with both TSS NDR relative coverage and TSS 2K region relative coverage greater than 1.5 in more patients were regarded as possessing closed chromatin states (Figure 3C; yellow dots indicate nonpermissive genes with closed TSS chromatin states). TCGA STAD RNA‐seq datasets were used to confirm that the permissive genes had significantly higher gene expression levels than the nonpermissive genes (Figure 3D). TSS NDR relative coverage and TSS 2K region relative coverage of the upregulated genes in the TCGA STAD RNA‐seq datasets had significantly lower coverage than those of the downregulated genes (Figure 3E–G). In addition, the TSS NDR relative coverage of healthy individuals had the opposite relationship with the RNA expression levels of PBMCs (Figure S5B). Furthermore, 794 genes of the top 1000 expressed genes in TCGA STAD RNA‐seq are predicated on the expressed status by TSS NDR relative coverage and TSS 2K region relative coverage in patients with gastric cancer (Figure S5C). GTEx PBMC RNA‐seq analysis revealed that 857 genes are predicted on the expressed status by TSS NDR relative coverage and TSS 2K region relative coverage in healthy samples (Figure S5D).

**FIGURE 3:**
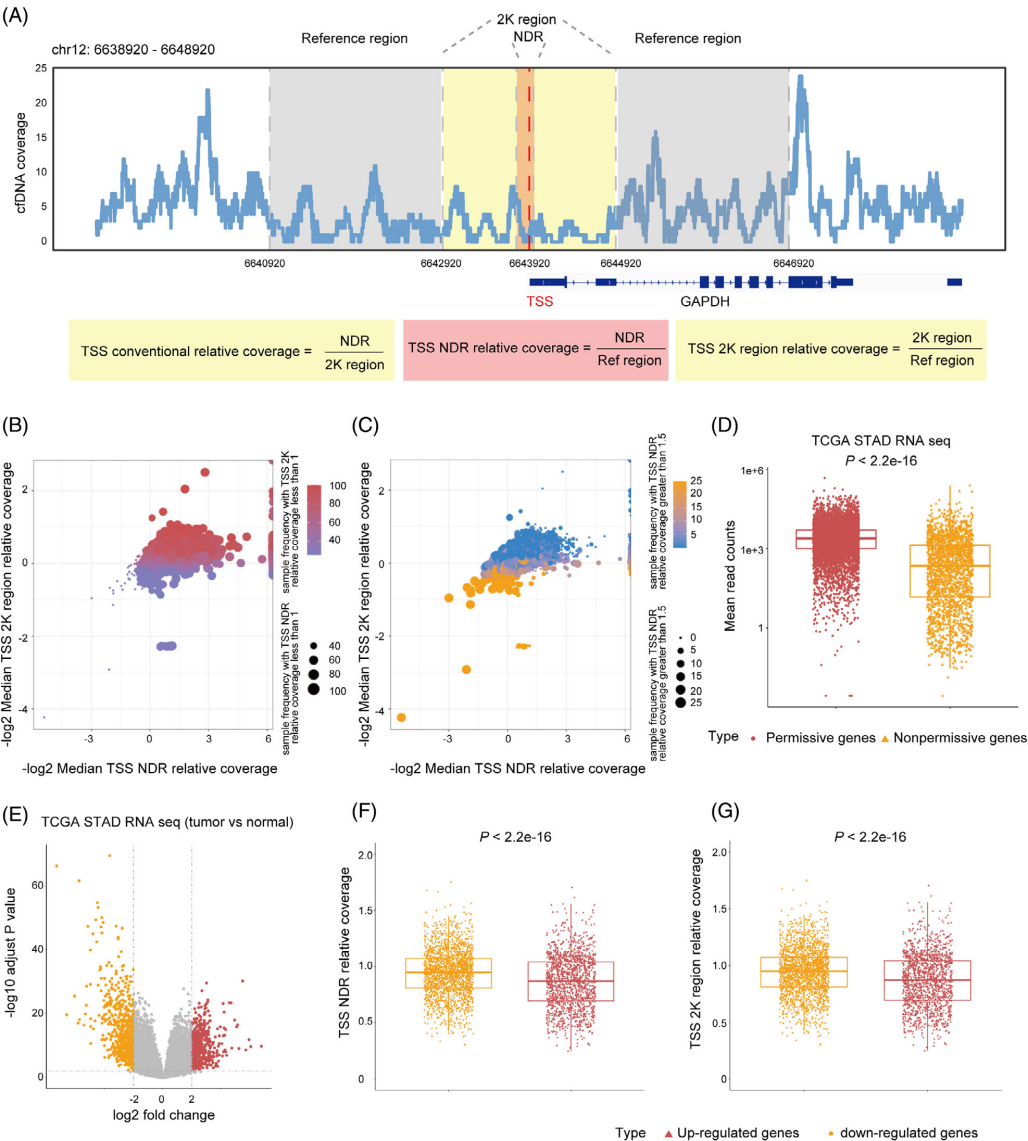
Integrated analysis toolkit for whole‐genome‐wide features of cell‐free DNA (INAC) TSS coverage indicates the dynamics of cancer transcriptome. (A) Integrative genetic viewer showing the NDR, 2K and reference region around TSS and the approaches used to calculate TSS conventional relative coverage, TSS NDR relative coverage and TSS 2K region relative coverage. (B) TSS NDR and 2K region relative coverage of gastric cancer samples showing the median value and frequency with TSS coverage less than 1. The dot size represents the sample frequency with TSS NDR relative coverage less than 1. Colour represents the sample frequency with a TSS 2K region relative coverage of less than 1. The x‐ and y‐axes show ‐log2 transformed values. (C) TSS NDR and 2K region relative coverage of gastric cancer samples showing the median value and frequency with a TSS coverage greater than 1.5. The dot size represents the sample frequency with a TSS NDR relative coverage greater than 1.5. Colour represents the sample frequency with a TSS 2K region relative coverage greater than 1.5. The x‐ and y‐axes show ‐log2 transformed values. (D) Boxplot showing the expression levels of the permissive and nonpermissive genes in the TCGA STAD RNA‐seq datasets. The two‐sided Wilcoxon test was used for the analysis. (E) Volcano plot showing differences in gene expression levels between tumour and normal tissues in the TCGA STAD RNA‐seq datasets. (F‐G), Boxplot showing the TSS NDR relative coverage (F) and TSS 2K region relative coverage (G) of the upregulated and downregulated genes in the TCGA STAD tumours.

Consistent with previous conclusions, the upregulated genes with high PFE had higher gene expression levels than the downregulated genes with low PFE (*p* = 1.4e‐8)^7^ (Figure S6A–C). These upregulated genes in the TCGA STAD RNA‐seq datasets had a higher mean PFE than the downregulated genes in the gastric cancer group or healthy group (Figure S6E, *p* < 2.2e‐16 in the gastric cancer group, *p* < 2.2e‐16 in the healthy group).

These features achieved good performance in the training and test datasets (Figure 4A–C). All features could achieve an area under the curve (AUC) of 0.91 by using a stochastic gradient descent algorithm (Figure S7B). However, 100 samples (50 patients with cancer and 50 healthy individuals) could not confirm the robustness and effectiveness of INAC, a large scale of samples is needed to estimate the performance of INAC. In addition, most of the gastric cancer samples and healthy samples had a consistent correct estimate based on the six cfDNA features. More than two cfDNA features predicted the wrong label in the same sample, but other cfDNA features could indicate the true label (Figure S7A). We also finished the transcription factor (TF) nucleosome occupancy maps in our cohort, these TFs could also distinguish patients with cancer from healthy individuals (Figure S7C,D). Furthermore, INAC also supported hundreds of machine learning methods to get the testing prediction accuracy through 10‐fold cross‐validation. Six cfDNA features could achieve the best AUC by using different methods (Figure S8A–F).

**FIGURE 4:**
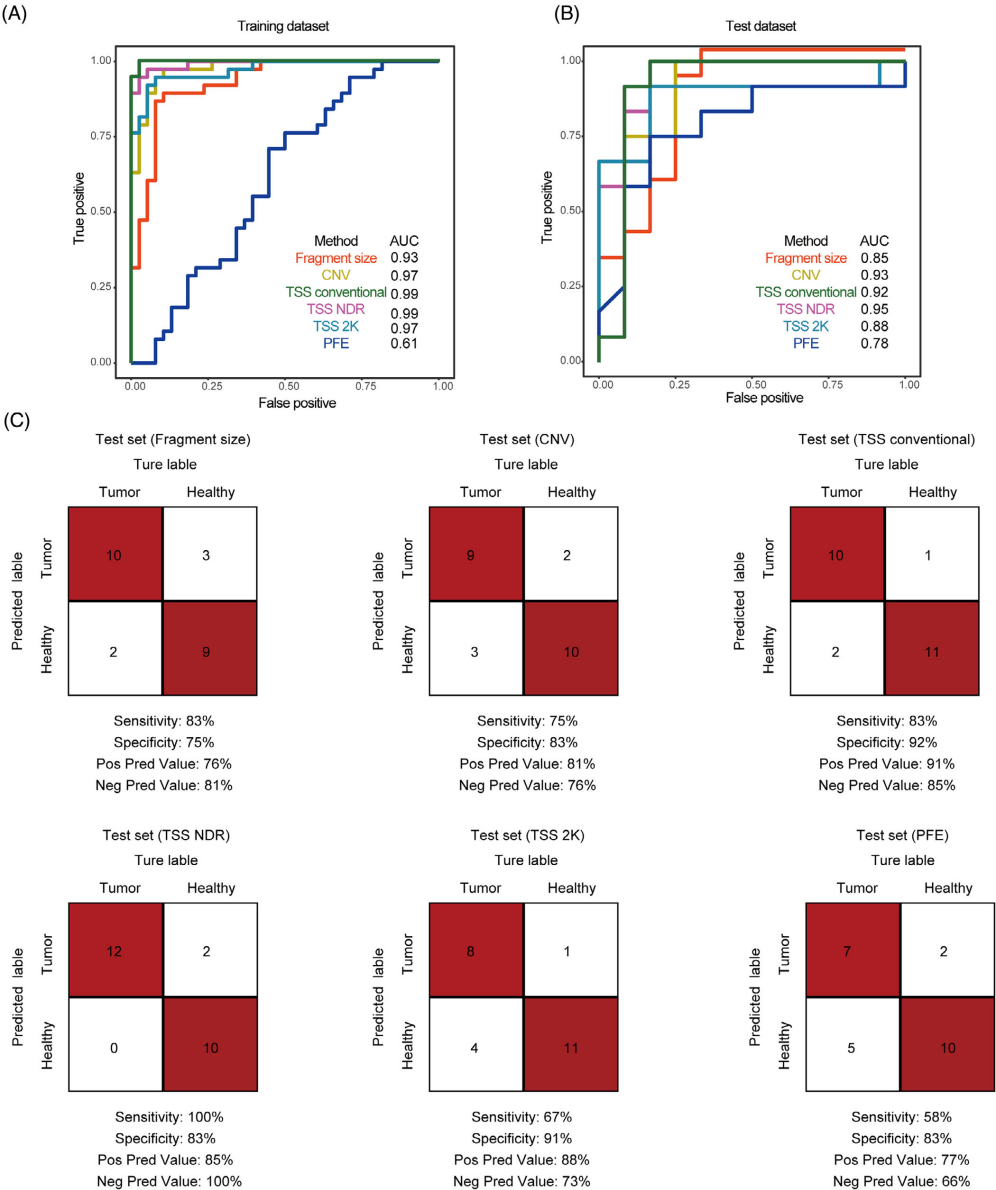
Integrated analysis toolkit for whole‐genome‐wide features of cell‐free DNA (INAC) multimodal machine learning supports cancer detection. (A, B) The performance of the receiver operating characteristic curve for detecting patients with gastric cancer using the five cfDNA features is shown in the training dataset (A) and test dataset (B). (C) Classification error matrix using the five cfDNA features in the test set.

In this study, INAC was shown to be able to assess whole‐genome‐wide features of plasma cfDNA.

## AUTHOR CONTRIBUTIONS

Jie Li, Zhaode Bu., Jiafu Ji. and Xun Lan. conceived this project. Jie Li. and Jiahui Chen. collected the patient blood and healthy blood. Jie Li. performed bioinformatics analysis. Xin Sun. performed the experiments. Jie Li. and Xun Lan. wrote the manuscript. All authors read or provided comments on the manuscript.

## CONFLICT OF INTEREST STATEMENT

The authors declare no conflict of interest.

## ETHICS STATEMENT

All blood samples were obtained according to a protocol approved by the Ethics Committee of Peking University Cancer Hospital (2020KT101).

## Supporting information

Supporting Information

Supporting Information

Supporting Information

Supporting Information

Supporting Information

Supporting Information

Supporting Information

Supporting Information

Supporting Information

Supporting Information

## Data Availability

The accession number for the sequencing data has been deposited in the Genome Sequence Archive under project PRJCA013939 (https://ngdc.cncb.ac.cn/gsa‐human/browse/HRA003821). The processed data of fragment size, CNV, TSS, PFE and TF has been deposited in OMIX002911 (https://ngdc.cncb.ac.cn/omix/view/OMIX002911). Codes were deposited in the Git hub: https://github.com/jacklee2thu/INAC.
