# Supplementary material for: Integrated analysis toolkit for dissecting whole‐genome‐wide features of cell‐free DNA
Source: Clin Transl Med. 2023 Feb 28;13(3):e1212. doi: 10.1002/ctm2.1212 (PMC9975452; doi:10.1002/ctm2.1212)
Supplement: Supplementary file 1 — Supporting Information [file CTM2-13-e1212-s001.docx]

**Supplement table 1. INAC supplied functions**

| **Function name** | **Type** | **Input files** | **main out** |
| --- | --- | --- | --- |
| INAC_initial_QC | QC | BAM | the mapped reads count, coverage, mean depth, mean MAPQ in 24 chromatin and mitochondria |
| INAC_QC | QC | BAM | the fraction of cfDNA fragment size on 30-80, 80-150, 150-220, 220-1000, 1000-longer (bp) |
| INAC_FR | Feature | BAM | the counts and fraction of short and long cfDNA fragments on whole genome wide |
| INAC_FR_visibility | visibility | feature matrix | the cfDNA fragments ratio on whole genome wide |
| INAC_CNV | Feature | BAM | the number of copy number variance of cfDNA on whole genome wide |
| INAC_TSS_conventional | Feature | BAM | the relative coverage of conventional region around TSS locations |
| INAC_TSS_2K | Feature | BAM | the relative coverage of 2K region around TSS locations |
| INAC_PFE | Feature | BAM | the PFE values around TSS locations |
| INAC_ML | visibility | feature matrix | model performance of each feature |
| INAC_TSS_NDR | Feature | BAM | the relative coverage of NDR around TSS locations |
