# Supplementary material for: Integrated analysis toolkit for dissecting whole‐genome‐wide features of cell‐free DNA
Source: Clin Transl Med. 2023 Feb 28;13(3):e1212. doi: 10.1002/ctm2.1212 (PMC9975452; doi:10.1002/ctm2.1212)
Supplement: Supplementary file 2 — Supporting Information [file CTM2-13-e1212-s003.docx]

**Supplement methods**

**Patient enrollment and sample collection**

We collected blood from 50 patients with gastric cancer and 50 healthy controls from Peking University Cancer Hospital. According to the exclusion criteria, any patients with gastric cancer who had previously been treated (including chemotherapy, radiation, or surgery), were pregnant, or had infections were excluded. Blood from healthy controls was donated from healthy volunteers. These volunteers with a negative screen for gastrointestinal disease and no recent pregnancy or serious infection were included in this research. The study was approved by the Ethics Committee of Peking University Cancer Hospital (2020KT101), and informed consent for use was obtained from all participants by the contributing institutions.

**Sample preparation and cfDNA sequencing**

Blood samples (~ 2 ml) were freshly collected from fasting patients using cfDNA collection tubes (cell-free DNA BCT, Streck, #218961). Plasma was separated by centrifugation at 2000 × g for 10 min at 4°C, centrifuged a second time at 2,000 g at room temperature to remove any remaining cellular debris, and then stored at −80°C until the time of DNA extraction. DNA was isolated from plasma using the Qiagen Circulating Nucleic Acids Kit (Qiagen, #55114 GmbH) according to the manufacturer’s instructions.

NGS cfDNA libraries were prepared for WGS using at least 5 ng of cfDNA as input material. Briefly, genomic libraries were prepared using the NEBNext Ultra II DNA Library Prep Kit for Illumina (New England Biolabs (NEB), #E7645S). Then, adapters with a single T overhang were ligated to the end-repaired dA-tailed fragments using Ultra II Ligation Master Mix and Ligation Enhancer. After excising U bases with the USER enzyme, the reactions were cleaned using SPRISelect beads (Beckman Coulter, # B23318), and DNA was eluted in 17 µl of H_2_O. For PCR, each reaction was mixed with 5 μL of 10 μM N5 primer, 5 µl of 10 μM N7 primer (NEB, #E7500S) and 25 µl of NEBNext Ultra II Q5 Master Mix. Amplification was performed using the following thermocycling program: 72°C for 5 min; 98°C for 30 sec; 12 cycles of 98°C for 10 sec, 63°C for 30 sec, and 72°C for 1 min; and a final incubation at 72°C for 5 min. PCR products were cleaned, and size ranges were assessed using an Agilent 2100 Bioanalyzer. Paired-end 150 bp sequencing was performed using the Illumina HiSeq Xten platform.

**Preprocessing of WGS cfDNA data**

The adapters of sequencing data were removed by TrimGalore software, and then paired-end reads of plasma samples were aligned to the human reference genome (version hg19) by Burrows–Wheeler Alignment Tool (bwa) software [^1^](#_ENREF_1). The mapped BAM files were sorted by SAM tools (version 1.9) [^2^](#_ENREF_2), and PCR duplicates were removed by Picard tools [“Picard Toolkit.” 2019. Broad Institute, GitHub Repository. http://broadinstitute.github.io/picard/; Broad Institute]. Next, reads with a MAPQ less than 30 were filtered. In addition, reads mapped into Duke blacklisted regions (http://hgdownload.cse.ucsc.edu/goldenpath/hg19/encodeDCC/wgEncodeMapability/) and with low map-ability in the hg19 reference genome were removed.

**cfDNA fragment size quality control**

Information on cfDNA fragment size was extracted from the processed BAM files. Then, these fragments were used to calculate the density of fragment sizes from 0 bp to more than 500 bp. The cfDNA fragment fractions from 30 bp to 80 bp, 80 bp to 150 bp, 150 bp to 220 bp, 220 bp to 1000 bp, and 1000 bp to longer were estimated in detail. These size ranges approximately corresponded to the length of cfDNA binding to a transcription factor, one nucleosome, one nucleosome plus a linker, and two or more nucleosomes.

**cfDNA fragment size ratio analysis**

The above extracted cfDNA fragment size information was used to finish cfDNA fragment size ratio analysis. First, the hg19 autosomes were divided into 504 adjacent, nonoverlapping 5 Mb bins. Fragments between 100 bp and 150 bp in length were defined as short, and fragments between 151 bp and 220 bp in length were defined as long.

To correct for coverage bias due to genomic GC content in 5 Mb bins, the function ‘loess’ from the R package ‘stats’ with default parameters (span setting of 0.75) was used to apply locally weighted scatterplot smoothing (LOESS) regression to calculate the average fragment GC content in each bin [^3^](#_ENREF_3). We calculated the predicted ratio of short and long coverage associated with the GC content and acquired the residuals for the proportion of coverage that was not associated with GC content. The resulting residuals plus the genome-wide median short and long estimates of coverage represent the GC-adjusted coverage in each bin.

Estimate values, including the fragment ratio (numbers of short fragments/numbers of long fragments) and scaled fragment ratio (normalizing the mean of the fragment ratio to zero in each sample), were used to compare the variability of fragment length between healthy samples and gastric cancer samples in 5 Mb bins.

**Analysis of variance and density of copy number in cfDNA**

A total of 51,120 adjacent, nonoverlapping 50 kb bins throughout the length of the genome were used to calculate cfDNA CNV scores [^4^](#_ENREF_4). Raw read counts per bin were adjusted for GC content by the above approach, and then we calculated the fraction of read counts in each bin. This fraction was normalized by the median fraction of healthy individuals and log2 transformed to obtain a copy number ratio per bin. Next, circular binary segmentation and hidden Markov model algorithms were used to separate the bins.

A cutoff of mean healthy CNV + 1.28*SD of healthy CNV was used to define the aberrant fraction of CNV. Amplified genes with the parameters ‘*P* < 0.05’ (Wilcoxon rank-sum two-tailed test, gastric cancer patient vs. healthy individual) and ‘log2 FC > 2’ (median CNV in gastric cancer patient vs. median CNV in healthy individual) were used to explore KEGG pathways and GO biological processes in gastric cancer.

**TSS coverage analysis of cfDNA**

TSS locations were searched in the ENSEMBL biomaRt database (hg19 version) (http://grch37.ensembl.org/index.html). Next, the SAM tools depth function was used to extract the coverage information around each TSS location from the processed BAM files. TSS NDR coverage was defined as 150 bp upstream to 50 bp downstream of the TSS, and TSS 2K region coverage was defined as 1000 bp upstream to 1000 bp downstream of the TSS. Subsequently, these two TSS coverages were normalized by the background region, and the combined regions contained coverage from 3000 bp to 1000 bp upstream with respect to the TSS and coverage from 1000 bp to 3000 bp downstream with respect to the TSS. Top 1000 expressed genes are predicted by TCGA STAD RNA-seq or GTEx PBMC RNA-seq. We calculate mean cfDNA TSS NDR relative coverage and cfDNA TSS 2K-region relative coverage of these genes in patients with gastric cancer or healthy samples. Expressed genes predicted by cfDNA TSS relative coverage are with value less than 1.

**PFE analysis of cfDNA**

The diversity of cfDNA fragment size around each TSS site (from 1000 bp upstream to 1000 bp downstream) was summarized as the Shannon entropy. The size ranges from 100 bp to 300 bp were defined as the bins (b_1_ = 100 bp to b_201_ =300 bp), and the density was estimated by the maximum likelihood. *P* = [p_1_, …, p_201_] with $P_{i}=\frac{n_{i}}{n}$, where $n_{i}$ represents the fragment number with length $b_{i}$ and n represents the total number of fragments at the TSS. Shannon entropy was defined as $-\sum p_{i} log2p_{i}$ [^5^](#_ENREF_5).

To adjust the batch effect of the sample sequencing depth or other bias, a Bayesian approach through a Dirichlet-multinomial model was used to finish the normalization step [^6^](#_ENREF_6). The bias from the sample and gene expression was considered as the two sides. For gene expression impact, two 250-bp regions within 1000 bp upstream to 750 bp upstream with respect to the TSS and 750 bp downstream to 1000 bp downstream were considered. For a sample, a sample-wide fragment length distribution containing the above two 250-bp regions using multinomial maximum likelihood estimation was calculated as a background distribution. For each TSS, the sample-wide background distribution was updated to calculate the gene-specific posterior and sample adjusted of the Dirichlet distribution based on fragment counts in the 201 size bins of the vicinity of the TSS: Dir (α* = α + [n1, …, n201]), the initial input parameter α of the Dirichlet distribution α0 = 20.

For Dir (α*), 2000 fragment length distributions were sampled, and the corresponding Shannon entropy was calculated. The calculated Shannon entropy was compared to the Shannon entropies of five randomly selected background gene sets, labeled e1, e2, e3, e4, and e5. Finally, PFE was defined as the likelihood of the gene-specific entropy exceeding the entropy of control background gene sets by (1+k) fold. This k satisfied a gamma distribution, k ~ Г (s = 0.5, r = 1). In summary, PFE(TSS) = Ek [$\sum_{i=1}^{5} p^{*} (e_{TSS}>\left( 1+k \right)* e_{i})$], where Ek[.] represents the expected value with the parameter k and $p^{*}$ represents the probability of the Dirichlet distribution Dir (α*) approximated by the 2000 draws.

**Transcription factor analysis from Ulz et al.**

Transcription factor pipeline published by Ulz et al. was from <https://github.com/PeterUlz/TranscriptionFactorProfiling>. The above processed hg19 aligned bam files and CNV files were taken into the script run_tf_analyses_from_bam.py to get transcription factor profiling on 1,000 sites for each transcription factor (TF). Subsequently, scoring pipeline was used to get High frequency amplitude (‘HighFreqRange’) for each of TF. TF profiling was deposited in https://ngdc.cncb.ac.cn/omix/preview/PK49l40P.

**Machine learning for cancer detection**

To distinguish gastric cancer patients from healthy individuals using the five identified cfDNA features (fragment ratio, CNV, TSS NDR relative coverage, TSS 2K region relative coverage, and PFE), we randomly divided the datasets into 75% training data and 25% test data and used a stochastic gradient boosting model (gbm) [^7^](#_ENREF_7). This approach in the training dataset estimated prediction error by tenfold cross-validation [^8^](#_ENREF_8). Next, all fragment size ratio features and the top dozens of other cfDNA features according to the importance index in the training dataset result were selected. All 504 features of fragment size ratio, top 50 features of CNV, top 100 features of TSS conventional relative coverage, top 50 features of TSS NDR relative coverage, top 100 features of TSS 2K region relative coverage, and top 100 features of PFE were selected by the feature importance rank in the training datasets through 10 tenfold cross-validation. A machine learning stochastic gradient boosting model was applied using the R package gbm with the parameters ‘interaction.depth = 3, n.trees = 150, shrinkage = 0.1, n.minobsinnode = 10’. The AUC was estimated in the training dataset and test dataset. The confusion matrix, sensitivity, specificity, positive prediction value, and negative prediction value were estimated in the test dataset.

**Differential expression analysis**

The R package ‘limma’ was used to perform the analysis of differential gene expression, TSS coverage and PFE, and the function ‘voom’ with the parameter ‘normalize="quantile"’ was used to preprocess the datasets [^9^](#_ENREF_9).

**Pathway and biological process enrichment analyses**

The function ‘enrichKEGG’ with the parameters ‘organism = "hsa", pvalueCutoff = 0.05’ and ‘enrich GO’ with the parameters ‘OrgDb=org.Hs.e.g..db, ont = "BP", pvalueCutoff = 0.05’ from the R package clusterProfiler were used to explore KEGG pathways and GO terms that were enriched among candidate genes [^10^](#_ENREF_10).

**REFERENCES**

1 Li, H. & Durbin, R. Fast and accurate long-read alignment with Burrows-Wheeler transform. *Bioinformatics* **26**, 589-595, doi:10.1093/bioinformatics/btp698 (2010).

2 Li, H. *et al.* The Sequence Alignment/Map format and SAMtools. *Bioinformatics* **25**, 2078-2079, doi:10.1093/bioinformatics/btp352 (2009).

3 Benjamini, Y. & Speed, T. P. Summarizing and correcting the GC content bias in high-throughput sequencing. *Nucleic acids research* **40**, e72, doi:10.1093/nar/gks001 (2012).

4 Scheinin, I. *et al.* DNA copy number analysis of fresh and formalin-fixed specimens by shallow whole-genome sequencing with identification and exclusion of problematic regions in the genome assembly. *Genome research* **24**, 2022-2032, doi:10.1101/gr.175141.114 (2014).

5 Shannon, C. E. The mathematical theory of communication. 1963. *M.D. computing : computers in medical practice* **14**, 306-317 (1997).

6 Yu, P. & Shaw, C. A. An efficient algorithm for accurate computation of the Dirichlet-multinomial log-likelihood function. *Bioinformatics* **30**, 1547-1554, doi:10.1093/bioinformatics/btu079 (2014).

7 Su, X. & Bai, M. Stochastic gradient boosting frequency-severity model of insurance claims. *PloS one* **15**, e0238000, doi:10.1371/journal.pone.0238000 (2020).

8 Parente, J. D., Chase, J. G., Moller, K. & Shaw, G. M. Kernel density estimates for sepsis classification. *Computer methods and programs in biomedicine* **188**, 105295, doi:10.1016/j.cmpb.2019.105295 (2020).

9 Ritchie, M. E. *et al.* limma powers differential expression analyses for RNA-sequencing and microarray studies. *Nucleic acids research* **43**, e47, doi:10.1093/nar/gkv007 (2015).

10 Yu, G., Wang, L. G., Han, Y. & He, Q. Y. clusterProfiler: an R package for comparing biological themes among gene clusters. *Omics : a journal of integrative biology* **16**, 284-287, doi:10.1089/omi.2011.0118 (2012).

**Figure S1. INAC quality control measurement of individual sample.** (A) cfDNA read counts are statistically described as all reads, deduplicated reads and human genome mapped reads in the hemolysis, healthy, and gastric cancer samples, respectively. (B) Bar plot showing the cfDNA human genome mapped reads of sequencing with paired-end in 24 chromosomes and mitochondria of the individual sample. (C-E). Bar plot showing individual sample cfDNA coverage (C), mean depth (D) and mean MAPQ (E) of sequencing with paired-end in 24 chromosomes and mitochondria.

**Figure S2. INAC fraction of GC-corrected short and long fragments.** (A-B) The fraction of GC-corrected short fragments (A) and long fragments (B) (defined as the fraction of fragments in the 5 Mb bin to all fragments in all bins) are described for gastric cancer samples (n=50) and healthy samples (n=50). (C-D) The Pearson coefficients of the fraction of GC-corrected short fragments (C) and the fraction of GC-corrected long fragments (D) showing the similarity of individuals from gastric cancer samples and healthy samples. (E) Boxplot showing the fraction of GC-corrected short fragment Pearson coefficients between the healthy median fragment ratio and each other sample. (F) Boxplot showing the fraction of GC-corrected long fragment Pearson coefficients between the healthy median fraction of GC-corrected fragments and each other sample. The two-sided Wilcoxon test was used for the analysis.

**Figure S3. INAC CNV captures tumor-derived programs. (A)** Plasma cell-free DNA copy number alteration shows the cfDNA CNV density of a gastric cancer sample (top) and a healthy sample (bottom). **(B)** Boxplot shows the fraction of abnormal cfDNA CNV in patients with gastric cancer and healthy individuals (groups were compared using the Wilcoxon rank sum test). **(C)** Scatter plot showing the distribution of the median CNV in gastric cancer samples and healthy samples. Red points represent significantly amplified cfDNA CNV in gastric cancer patients compared with healthy individuals (Wilcoxon rank-sum two-tailed test *P* <0.05 and fold change >=2). **(D)** Gastric cancer-related KEGG pathways and GO biological processes enriched based on the above amplified genes.

**Figure S4. Aberrant CNV captures tumor-derived programs.** (A) Boxplot shows the cfDNA CNV in patients with gastric cancer and healthy individuals (groups were compared using the Wilcoxon rank sum test). (B) Heatmap showing the cfDNA CNV values of amplified genes and corresponding bins involved in the indicated pathways.

**Figure S5. INAC TSS coverage indicates the dynamics of cancer transcriptome.** (A) Heatmap showing the TSS conventional relative coverage, TSS NDR relative coverage and TSS 2K region coverage in 50 patients with gastric cancer. (B) Scatter plot showing the relationship between healthy sample cfDNA TSS NDR relative coverage and PBMC RNA TPM levels. The x-axes show -log2 transformed values. (C-D) Venn plots show the overlap between top 1000 expressed gene predicted by TCGA STAD RNA-seq (C) or GTEx PBMC RNA-seq (D) and cfDNA TSS relative coverage.

**Figure S6. INAC PFE infers gene expression levels. (A)** Scatter plot showing the relationship between healthy sample cfDNA PFE and PBMC RNA TPM levels. The x-axes show -log2 transformed values. **(B)** Volcano plot showing differences in cfDNA PFE between gastric cancer samples and healthy samples. **(C)** Boxplot showing TCGA STAD RNA-seq mean read counts of the upregulated and downregulated genes that were identified based on the PFE in gastric cancer samples and healthy samples. The two-sided Wilcoxon test was used for the analysis. **(D)** Boxplot showing the mean PFE of the upregulated genes and downregulated genes in gastric cancer samples and healthy samples, respectively.

**Figure S7. Confusion heatmap and Transcription factors profiling.** (A) Confusion heatmap showing the performance of the five cfDNA features in distinguishing gastric cancer samples and healthy samples. (B) The performance of the receiver operating characteristic curve for detecting patients with gastric cancer using all features based on the five machine learning methods is shown in the test dataset. (C) the volcano plot showing the different TF between patients with gastric cancer and healthy individuals. (D) The performance of the receiver operating characteristic curve to detect patients with gastric cancer using TF is shown in the test dataset.

**Figure S8. Performance of cancer detection based on different machine learning methods. (A-F)** The performance of the receiver operating characteristic curve for detecting patients with gastric cancer using fragment ratio (A), CNV (B), TSS NDR relative coverage (C), TSS 2K region relative coverage (D), TSS conventional relative coverage (E) and PFE (F) based on gbm, glm, random forest, svm linear and dwd linear is shown in the test dataset.
