# Supplementary figures and images for: Integrated analysis toolkit for dissecting whole‐genome‐wide features of cell‐free DNA
Source: Clin Transl Med. 2023 Feb 28;13(3):e1212. doi: 10.1002/ctm2.1212 (PMC9975452; doi:10.1002/ctm2.1212)

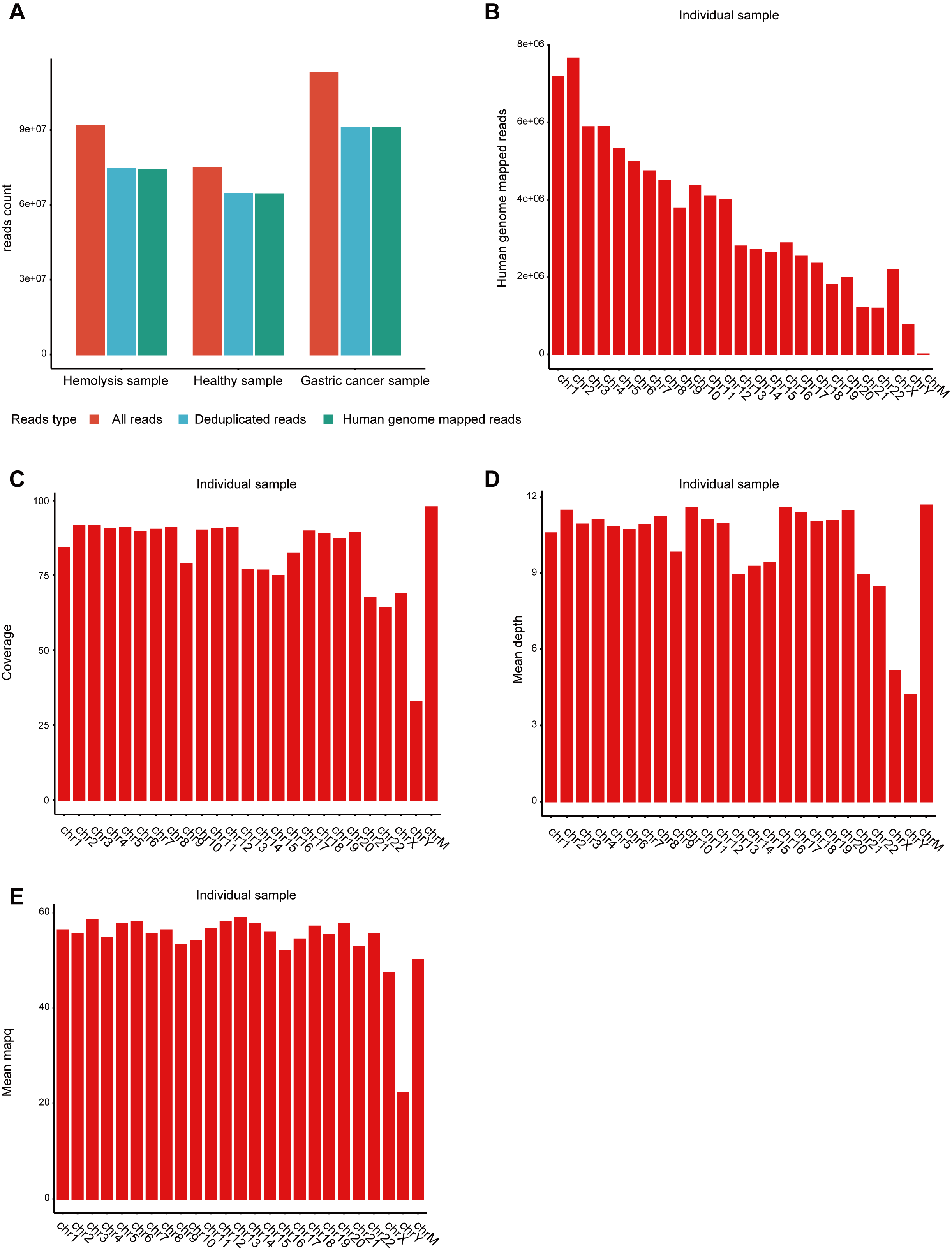

Supplement: Supplementary file 3 — Supporting Information [file CTM2-13-e1212-s005.tif]

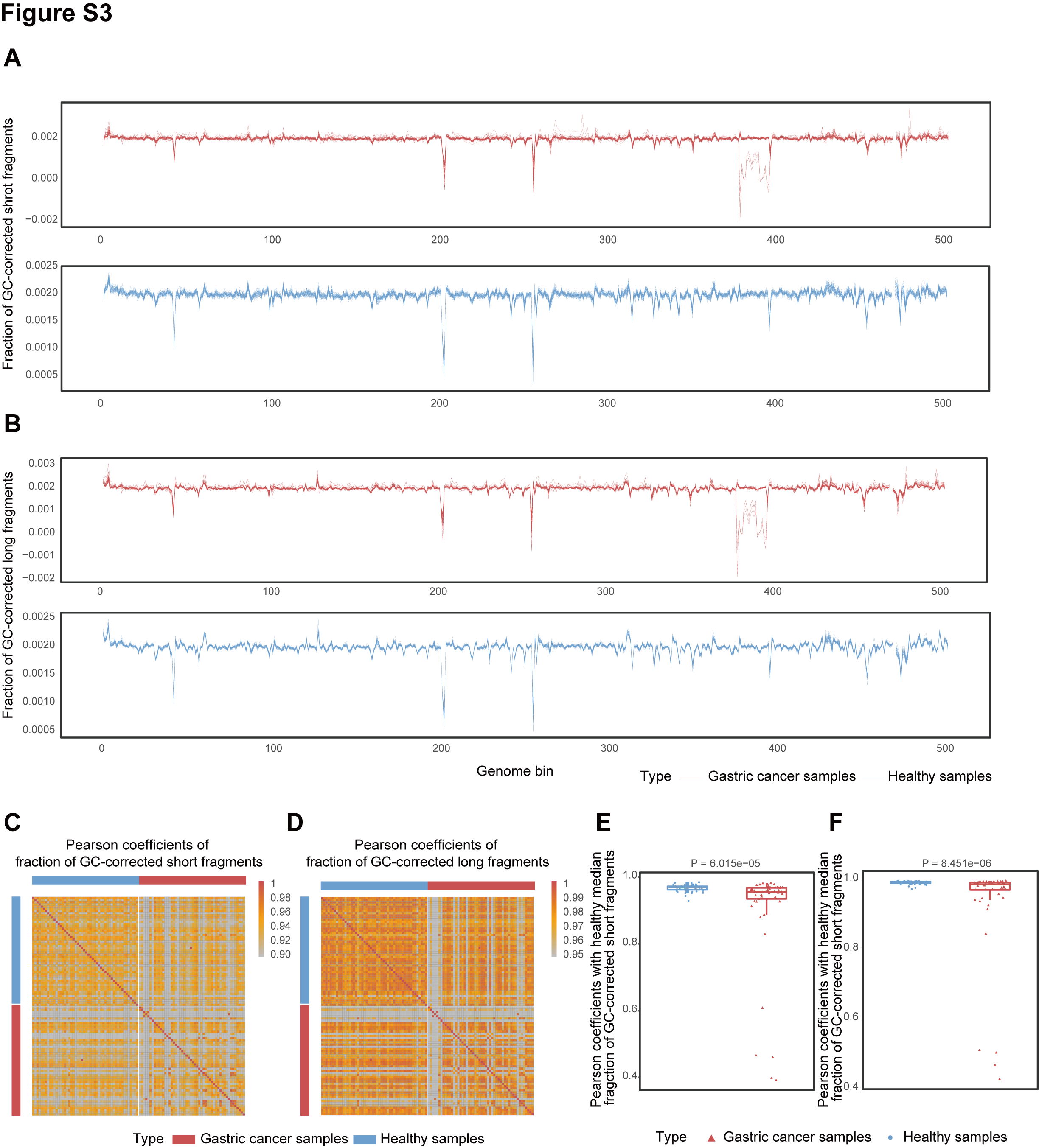

Supplement: Supplementary file 4 — Supporting Information [file CTM2-13-e1212-s004.tif]

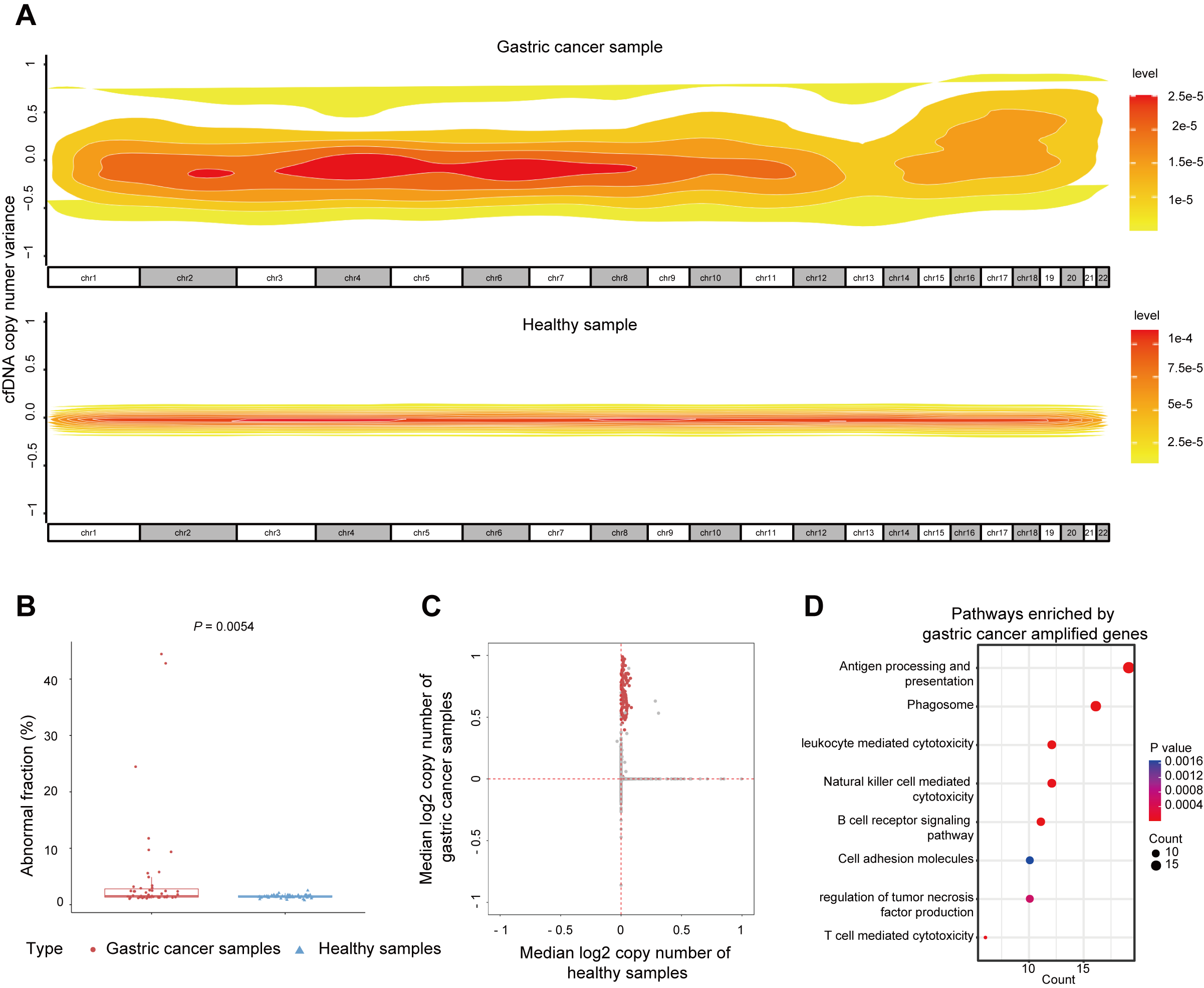

Supplement: Supplementary file 5 — Supporting Information [file CTM2-13-e1212-s002.tif]

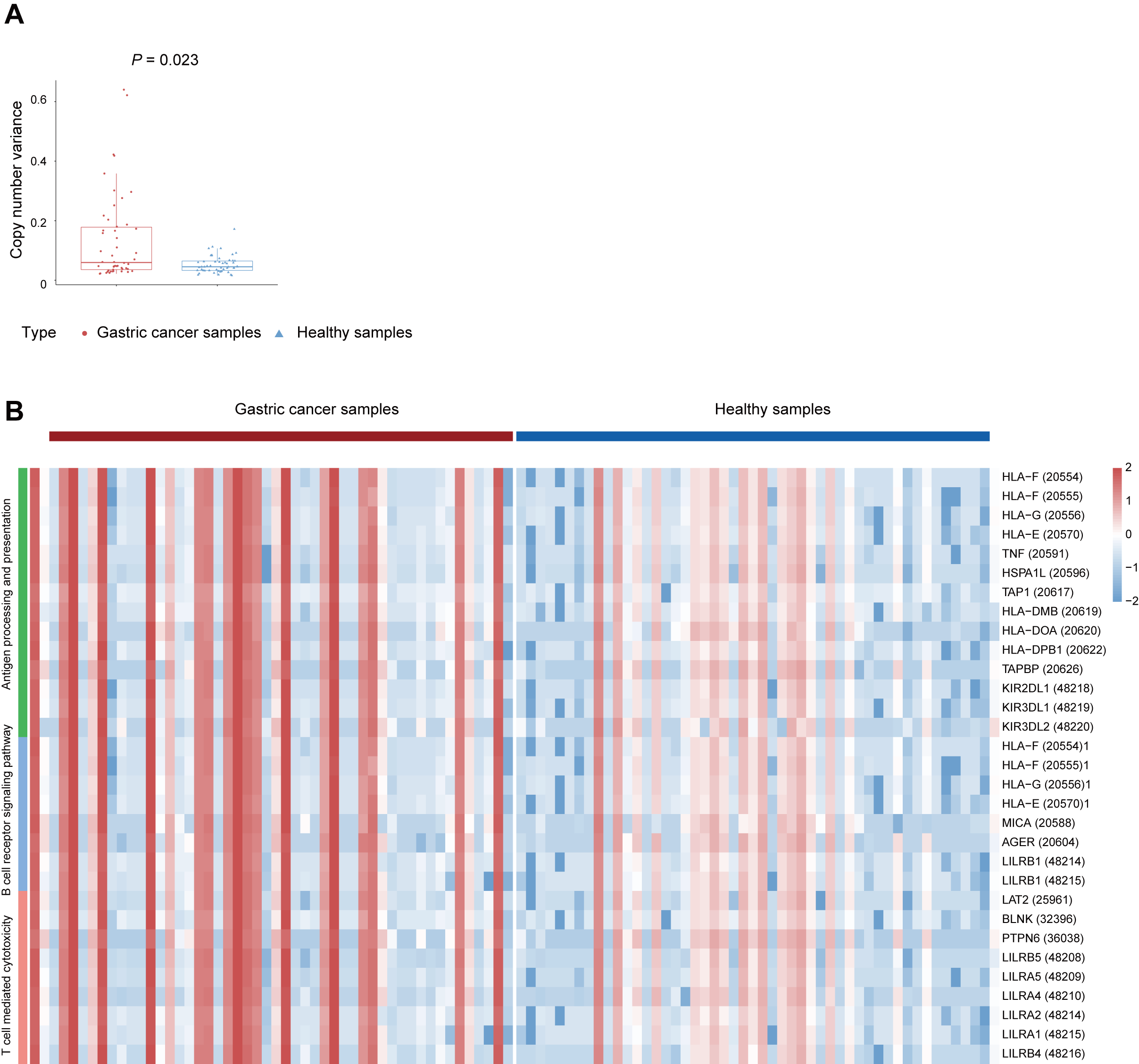

Supplement: Supplementary file 6 — Supporting Information [file CTM2-13-e1212-s009.tif]

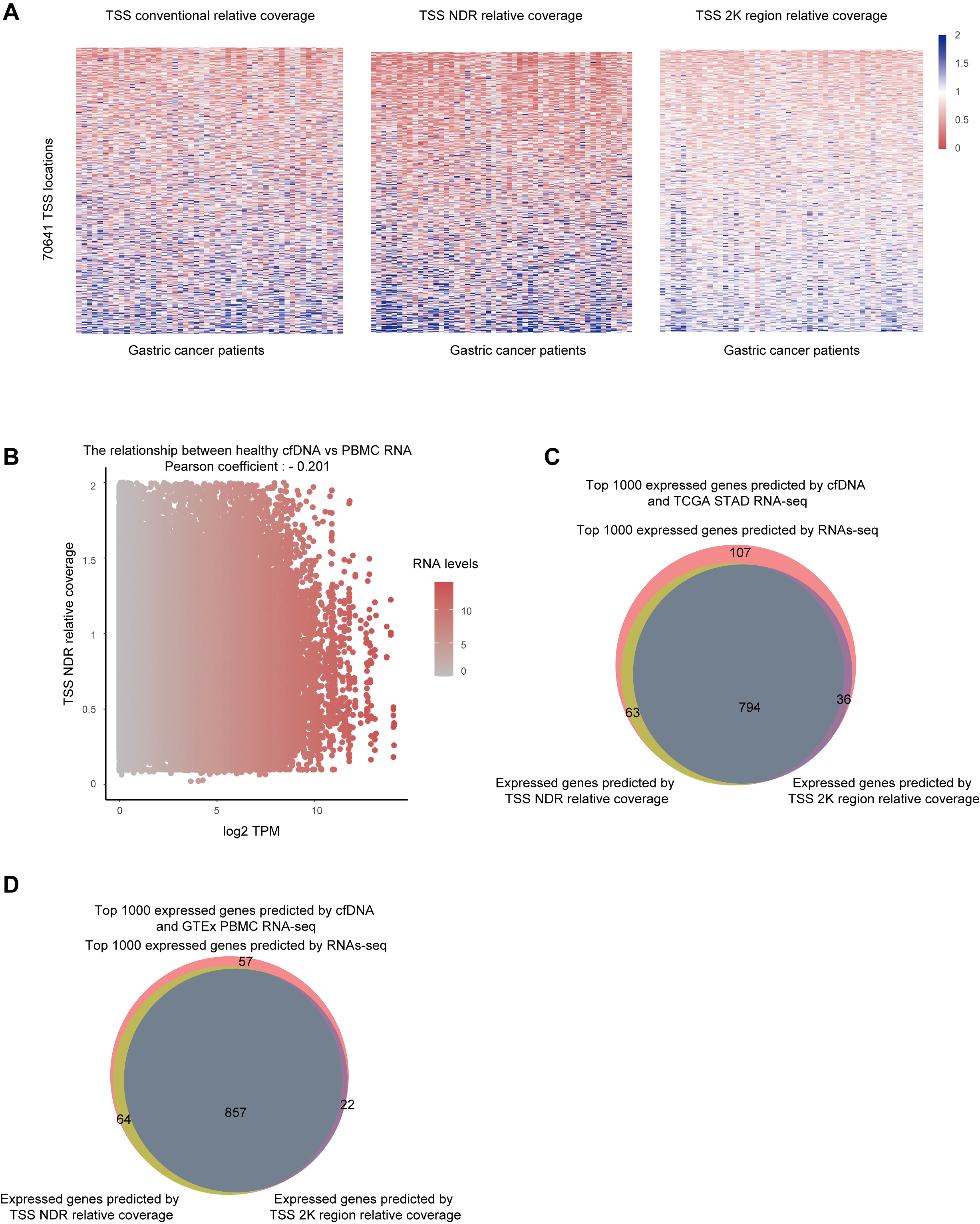

Supplement: Supplementary file 7 — Supporting Information [file CTM2-13-e1212-s006.tif]

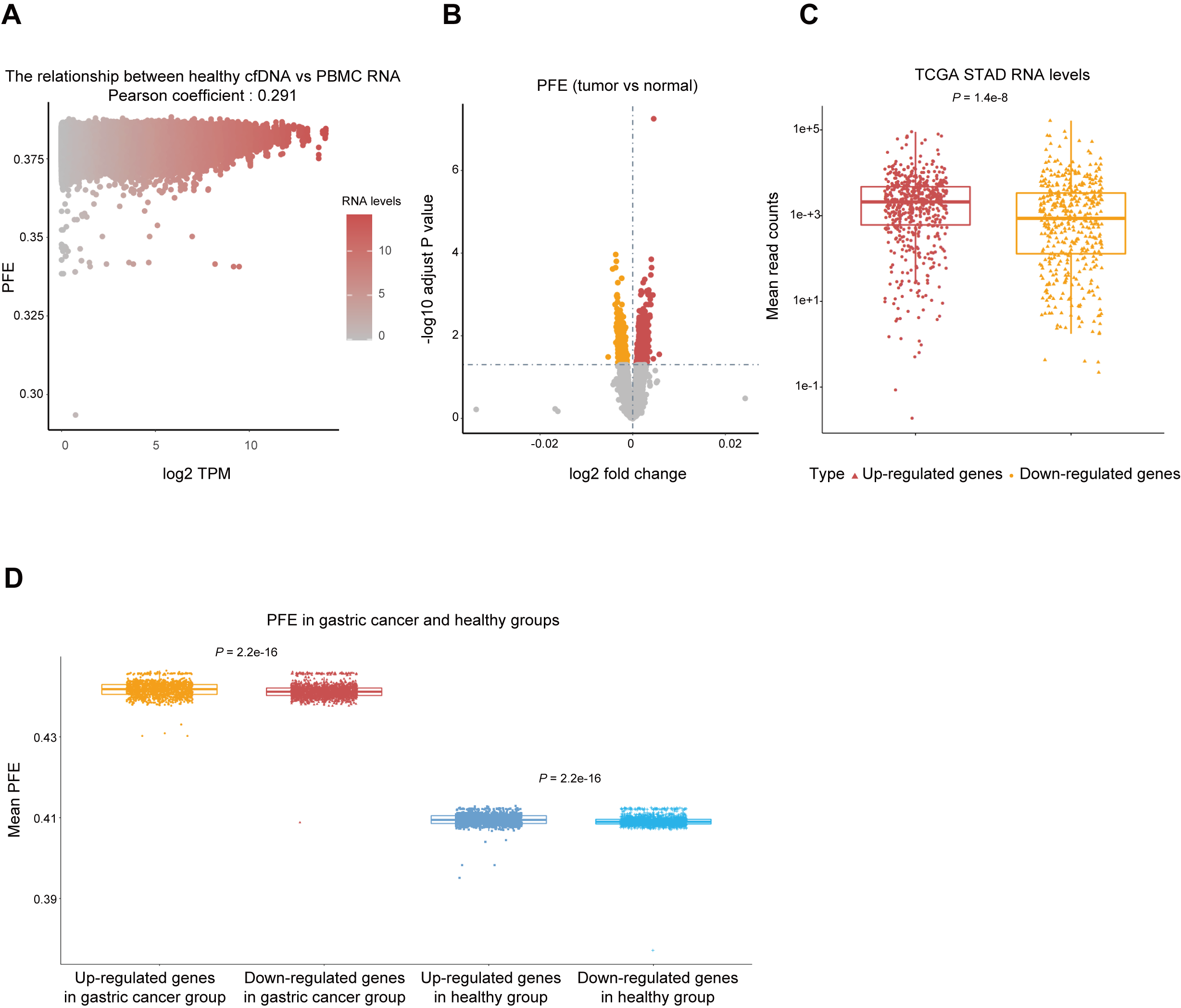

Supplement: Supplementary file 8 — Supporting Information [file CTM2-13-e1212-s007.tif]

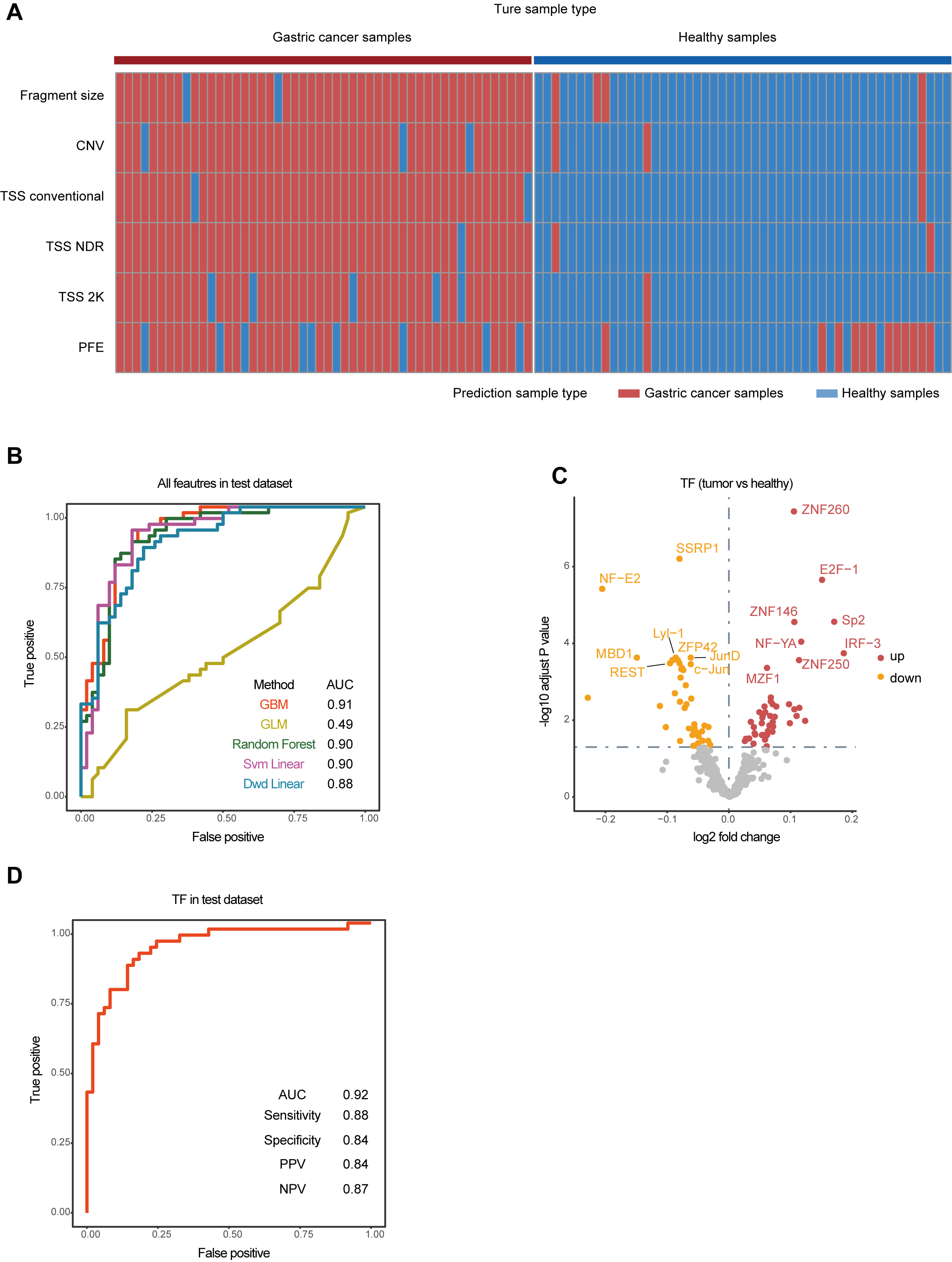

Supplement: Supplementary file 9 — Supporting Information [file CTM2-13-e1212-s010.tif]

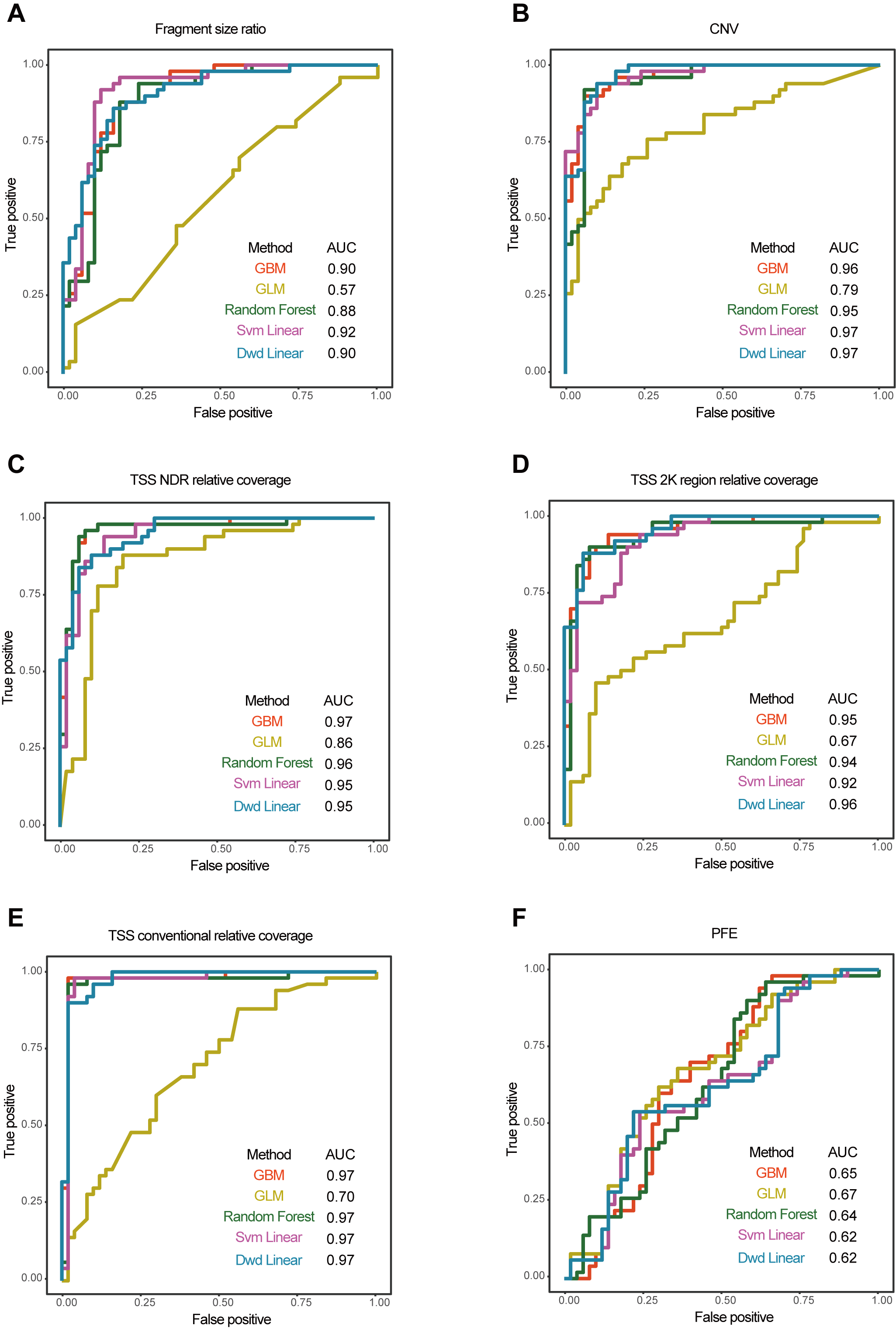

Supplement: Supplementary file 10 — Supporting Information [file CTM2-13-e1212-s008.tif]
